# The Surreptitious Abolition of Compulsory Vaccination

**Published:** 1907-10-26

**Authors:** 


### PUBLIC HEALTH AND HYGIENE.

### "THE SURREPTITIOUS ABOLITION OF COMPULSORY VACCINATION."

SUCH was our description of the Vaccination Act of 1907 while it was yet in the stage of a Bill before Parliament. Of its proposals we said: "It is impossible to escape the conclusion that the effect will be to make vaccination in the future more honoured in the breach than the observance," while of the strategy displayed in promoting the measure we pointed out that it was "a tribute to the mastery of finesse of the astute President of the Local Government Board that, with the passage of his Bill, he would have effectually abolished, almost without opposition, the compulsory character of vaccination in England and Wales."

We were convinced that the solid body of opinion in this country, both expert and lay, which affirmed the necessity of preserving the compulsory character of vaccination, bade fair to be outflanked by the tactics of the anti-vaccinists in thus giving legislative effects to views which, though widely held, were yet the irresponsible craze of a noisy and ignorant minority.

Sir John Batty Tuke and Sir Henry Craik, if we remember right, made a valiant stand of opposition to the measure, yet the fact remains that one of the most momentous enactments of the present Government slipped through Parliament without anyone apparently being aware of the sweeping character of this most dangerous measure. Ask the man in the street to-day whether vaccination be compulsory in this country, and he will answer, "Yes, of course. A few fanatics can get exemption by some new-fangled process of conscientious objection, but it is compulsory for all ordinary citizens."

The man in the street did not read the Vaccination Act of 1907, and it is not until he registers the birth of his next child that he will learn that the Local Government Board has provided him with official forms, the filling-in of which will exempt him from all the trouble and expense of vaccination, a practice which his wife has always insisted was responsible for every ailment of every other member of his family. Officially encouraged, who will wonder that he should purchase immediate domestic peace by construing his conscience in accord with the presiding spirit of his household? The new vaccination order which provides for this simple escape is as follows:—

NOW THEREFORE, in pursuance of the powers given to Us by the Statutes in that behalf, We hereby Order and Direct that, from and after the First day of October, One thousand nine hundred and seven, Article 30 of the Order, and the Fifth Schedule thereto, shall apply and have effect as if the Form of Notice, which is in that Article referred to as "Form A" and which is hereinafter referred to as "Form A, Part I," were further modified by the addition thereto of the following words and figures (hereinafter referred to as "Form A, Part II"); that is to say:

FORM A, PART II,

**To be given by the Registrar of Births (together with Form A, Part I) to the Person to whom notice is required to be given under Section 15 of the Vaccination Act, 1867, in the case of all Children born after the 31st day of August, 1907.**

**On and after the First day of January, 1908, exemption from penalties under Section 29 or Section 31 of the Vaccination Act, 1867,** will not be obtainable in the manner referred to in paragraph 6 of Form A, Part I, but on and after that date you will be exempt from any penalty under either of those sections for not having the child vaccinated if, within four months from the birth of the child, you make in the Form set out below, or in a Form to the like effect, a statutory declaration of your conscientious belief that vaccination would be prejudicial to the health of the child, and within seven days thereafter deliver the declaration, or send it by post, to the Vaccination Officer of the District whose name and address are given on the sheet containing Form A, Part I.

FORM OF DECLARATION.

I, , of in the parish of in the county of , being the parent [*or* person having the custody] of a child named , who was born on the day of , 19 , do hereby solemnly and sincerely declare that I conscientiously believe that vaccination would be prejudicial to the health of the child, and I make this solemn declaration conscientiously believing the same to be true, and by virtue of the provisions of the Statutory Declarations Act, 1835.

Dated this day of , 19 .

(Signed)

Declared before me, at , on the day of .

a Commissioner for Oaths [*or* Justice of the Peace, *or* other officer authorized to receive a statutory declaration].

At an estimated cost of 1s. and the filling in of the "Form of Declaration" actually thrust into his hands by a Government official acting under Government orders, he will escape all further trouble about vaccination. And this form, be it observed, is one which may be subscribed by every one, no matter how convinced he may be of the value of vaccination. It is of the very essence of vaccination that it is "prejudicial to the health of the child."

Everyone knows that vaccination is the intentional transmission of a disease in order that an infinitely worse condition may be escaped, and every medical man in the country may solemnly and sincerely subscribe to his conscientious belief that whatever benefits it may ultimately confer vaccination will immediately "be prejudicial to the health of the child."

So much has been accomplished by the anti-vaccinist, while the Government retains the shame of appearing to conform to the universal verdict of authoritative opinion as to need for compulsory vaccination if small-pox is to be kept at bay. The fruit of this folly no doubt will be a calamitous visitation of small-pox. A few years will see an enormous growth of populations unprotected from small-pox, and it is then that we shall be faced, according to all the teaching of experience, with the devastations of this terrible disease.

It is to be feared, however, that nothing short of a sharp lesson thus taught will convince the people of this country that they cannot afford to ignore the authority of expert opinion. This latest edict of the Local Government Board deliberately ignores all authoritative pronouncements on the question of compulsory vaccination, but the responsibility rests with Parliament, and ultimately with the people of this country, who can consent to such flagrant and even cynical flouting of expert findings on this question.

